# The Welfare of Traveller and Gypsy Owned Horses in the UK and Ireland

**DOI:** 10.3390/ani12182402

**Published:** 2022-09-13

**Authors:** Marie Rowland, Neil Hudson, Melanie Connor, Cathy Dwyer, Tamsin Coombs

**Affiliations:** 1 Royal (Dick) School of Veterinary Studies, Easter Bush, Midlothian EH25 9RG, UK; 2 SRUC, Roslin Institute, Easter Bush, Midlothian EH25 9RG, UK; 3 Institute of Development Studies, Library Road, Brighton BN1 9RE, UK

**Keywords:** equine, travellers, gypsies, welfare assessment, emotional state, qualitative behaviour assessment

## Abstract

**Simple Summary:**

Travellers and Gypsies are recognised ethnic groups in the UK and Ireland. Horse ownership is an important part of their lives; however, poor horse welfare is often perceived to be associated with these horse-owning communities. Nevertheless, studies on the welfare of Traveller and Gypsy-owned horses are lacking. The welfare of 104 horses in the UK and Ireland was evaluated using a horse welfare protocol that assessed health conditions, resource provision, management and horse behaviour. In order to potentially understand how a horse was feeling, Qualitative Behaviour Assessment (QBA) was used to evaluate their body language. Most horses were found to have good body condition, a healthy coat and few skin problems or joint issues, however, 27% of horses were found to have neglected hooves. In the voluntary animal approach test, most horses showed a friendly response. Positive QBA terms were more prevalent than negative terms, therefore, the emotional state of Traveller and Gypsy owned horses was deemed to be positive overall. An association between QBA and various horse welfare measures was identified, e.g., improved mood was associated with better water availability. Findings in this study did not support previous negative perceptions of horse welfare in Traveller and Gypsy horse owning communities.

**Abstract:**

Travellers and Gypsies are recognised ethnic groups in the UK and Ireland. Horse ownership is an important cultural tradition, however, practices associated with poor welfare are often perceived to be linked to these horse owning communities. Despite this, empirical studies on the welfare status of Traveller and Gypsy owned horses are lacking. To determine the welfare status of Traveller and Gypsy owned horses, 104 horses were assessed using a bespoke horse welfare protocol. This protocol assessed animal, resource and management-based measures. In addition, Qualitative Behaviour Assessment (QBA) identified horses’ emotional state. Results indicated that 81% of horses had an optimal body condition score, with no horse recorded as very thin/fat. The absence of limb conditions (95%), ocular (98%) and nasal (93%) discharges were evident in most horses, and 81% of horses responded positively to the voluntary animal approach test. The most commonly observed welfare issues were hoof neglect (27%), with hoof cracks/breakages (19%) being the most prevalent. QBA indicated that positive emotional states were more commonplace than negative. A relationship between QBA and other horse welfare measures was observed, e.g., improved mood was associated with better water availability. This research provides novel data in the under-researched area of the welfare of Traveller and Gypsy owned horses and counters perceptions of a poor welfare state in this group of horses.

## 1. Introduction

The horse industry in the UK and Ireland is significant, with 847,00 horses recorded in Britain [1], and the most recent figures record approximately 159,000 in Ireland [2]. Today, the role of the horse is primarily for use in the sporting and recreational industries, with these roles ranging from elite sports to leisure activities. The horse is also central to Traveller and Gypsy communities. While Travellers and Gypsies have different origins, there are many similarities between these two groups, with a close-knit community, the extended family and family networks being of great importance. Fundamental to Traveller and Gypsy identity is their nomadic lifestyle, and central to this is the horse. Today, this lifestyle is now more likely to alternate between nomadic and static [3].

### 1.1. Traveller and Gypsy Horse Culture

Coloured Cobs and Vanners, also known as the Gypsy or Irish Cob, were the original Traveller and Gypsy horses and remain associated with Traveller/Gypsy horse ownership today. These horses were used to pull bow top wagons, which were the traditional Traveller and Gypsy accommodation. Horses also had a strong economic importance for Travellers and Gypsies as they enabled the nomadic nature of their economy [4]. Traditional skills and trades such as blacksmithing, coopering and field labouring were all reliant on horse drawn accommodation throughout rural communities. The horse drawn days came to an end in the 1960s, when motorised vehicles, rather than the horses, transported trailers around the country [5]. Further, with industrialisation, the migration of people from rural to urban areas also included the movement of Travellers and Gypsies to cities and towns. Nevertheless, horse ownership remains commonplace within the Traveller and Gypsy culture [4]. Today, Travellers and Gypsies keep, breed and sell horses, with horse dealing considered an important Traveller and Gypsy activity [4,6]. Traditional recreational activities include trotting and sulky racing [7]. Sulky racing is equivalent to harness racing, where a cart or sulky is pulled by the horse and controlled by a driver, and horses race at a specific gait (a trot or a pace).

At traditional horse fairs, Traveller and Gypsy horse owners trade and show horses. Some of the most well-known fairs include Appleby and Stowe in England and Ballinasloe in Ireland. Horse fairs date back centuries; for example, Appleby Horse Fair is the largest horse fair in Europe and dates back to 1775 [8], and it is considered one of the most important occasions in the Traveller and Gypsy calendar [9]. At these annual gatherings, Travellers and Gypsies celebrate their history and folklore, socialise, buy traditional wares and goods, and trade and barter in horses [10].

### 1.2. Horse Welfare

The limited literature identifies Traveller horses as being particularly vulnerable to reduced welfare [11,12,13], with practices such as fly-grazing, tethering, abandonment of animals and indiscriminate breeding being seen as common [14]. However, there is a lack of empirical data to support these views. As previously discussed, Traveller and Gypsy horse breeders, buyers and sellers also converge at annual horse fairs to trade in horses and, during these fairs, horses are often shown off on the ‘Flashing Lane’. The ‘Flashing Lane’ is an iconic part of the Appleby Horse Fair and, to a lesser extent, at Ballinasloe Horse Fair in Ireland, and it requires that a portion of road be cordoned off so horses can be ridden or driven at fast speeds. The purpose of the ‘Flash’ is to showcase horses, with horse dealers, buyers, sellers and spectators having the opportunity to assess a horse’s speed, health, temperament and beauty [15]. However, the riding and driving practices on the ‘Flashing Lane’ at Appleby horse fair have raised concerns for horses’ welfare [16], although there is a lack of robust information on the extent of these welfare issues. Therefore, the welfare of horses belonging to Traveller and Gypsy communities needs to be assessed to address this gap in our knowledge.

Equine welfare should be assessed using a welfare assessment protocol which may include animal, resource and management-based measures. According to Main et al., the status of the animal is best assessed using animal-based health and behaviour measures [17]. An important aspect of a welfare assessment protocol is behavioural measurements which can be used to understand an animal’s affective state. An innovative scientific approach used to measure the expressive quality of an animal’s behaviour and affective state is the ‘Qualitative Behaviour Assessment’ (QBA) [18]. QBA is a ‘whole animal approach’ and is used to measure how the animal is expressing behaviour, often referred to as an animal’s ‘body language’. An animal’s mood or emotional state is communicated through its body language and is assessed using terms that describe their emotional repertoire. This is then applied to interpret an animal’s physical and psychological state. According to Wemelsfelder, the animal’s welfare status is directly related to the quality of their expressions; for example, are animals’ content and playful or are they fearful or bored? [19]. QBA has been used successfully in a variety of species (cattle, [20]; horses, [21]; donkeys, [22]). QBA is inexpensive and user-friendly, and its inter-observer reliability and biological validity has been assessed [23,24,25]. Further, QBA is effective in detecting subtle differences in an animal’s body language and is valuable in the assessment of positive features of the animal’s status [22]. However, QBA is best used in conjunction with already established welfare protocols [25]. QBA was used in this study to support other welfare assessment measures.

The welfare status of Traveller and Gypsy owned horses is deemed to be poor, although there are few empirical studies supporting this perspective. Therefore, this study aims to identify the welfare status of Traveller and Gypsy owned horses and to determine the factors that are considered risks to horse welfare in this community. Traveller and Gypsy owned horses are often managed differently to horses in other communities, therefore, assessments of horses in situations such as fly-grazing, while tethered and at horse fairs was required. Further, this study aimed to develop a QBA fixed term list and describe the dimensionality of QBA in these horses.

## 2. Materials and Methods

This study was approved by the Human Ethics Review Committee (ref no. HERC_180_18) and Veterinary Ethics Review Committee (ref no. VERC_ 3_18), Royal (Dick) School of Veterinary Studies, University of Edinburgh.

### 2.1. Development of Welfare Assessment Protocol for Traveller and Gypsy Owned Horses

None of the existing protocols were deemed absolutely suitable for assessing the welfare of horses in the Traveller and Gypsy communities. Therefore, a bespoke protocol was developed. This tool focused on measures that were relevant to Traveller and Gypsy owned horses and included animal, resource and management-based measures.

Selection of welfare indicators*:* Measures from established equine welfare assessment tools were selected. Seventeen animal and resource-based indicators were considered for their relevance and value to equine welfare as well as its suitability for use with equines in this study. Due to the housing situations in which Traveller and Gypsy owned horses may be found, e.g., tethered, free roaming and at horse fairs, it was agreed that assessments would only include indicators that could be visually assessed.

Development of Qualitative Behaviour Analysis (QBA) terms*:* To assess the suitability of QBA as a tool to assess the welfare of Traveller and Gypsy owned horses, a focus group on QBA term generation in horses took place in Ireland in December 2017 with 6 Traveller horse owners. Irish Traveller horse owners were recruited at this early stage of the study because of their participation in previous research with a member of the research team. These horse owners did not participate in any other aspect of this study. An introduction to the QBA method of assessing animal behaviour was presented to participants and followed with a list of 12 behavioural expression descriptors, devised from existing equine QBA literature [21,22,26]. Participants reviewed and discussed this list of descriptors, agreeing on broad characterisations, and considered situations of when different descriptors could be used. Participants were also invited to include their own QBA terms, resulting in an additional ten terms and examples of their use. In the second session, participants refined five of the descriptors and eliminated seven terms that they believed were similar or difficult to interpret. Participants discussed and agreed on the definitions of each term. A fixed list of 15 descriptors and definitions of these descriptors were agreed on. The QBA tool was piloted on three horses, resulting in the elimination of a descriptor due to its similarity to another, with the final QBA tool consisting of fourteen QBA descriptors, Table 1.

The QBA descriptors were uploaded onto a tablet (Lenovo Tab 2 A10-70, Lenovo Group Limited, TAB 2 A10-70F ZA000015GB Tablet) where each behavioural expression descriptor was presented next to a visual analogue scale (VAS). The left end of the VAS represented the minimum score, indicating that the descriptor was absent in horses, and the right end represented the maximum score whereby the descriptor was highly present in horses. A vertical line was drawn across the VAS at the point that best represented the expressive quality, as indicated by the descriptor terms, of each horse assessed. Scoring of the behavioural expressions was recorded on the tablet.

Field testing the horse welfare assessment pilot protocol*:* The protocol was tested on ten non-Traveller and Gypsy horses in Scotland and England in March 2018. This revealed that all measures were relevant and practical to observe, Table 2.

### 2.2. Welfare Assessment Protocol

The protocol contained 17 measures in addition to demographic information. All indicators were visually assessed, and measures were scored either numerically, present/absent, yes/no or tick if applies (Table 2).

### 2.3. Data Handling

Data were recorded using the data collection tool ‘Kobo Toolbox’, an online/offline free software programme developed by the Harvard Humanitarian Initiative (https://www.kobotoolbox.org/, accessed on 1 April 2018). All horse welfare data collection was conducted by the main researcher.

### 2.4. Procedure

Horse owners were recruited through convenience and snow-ball sampling, starting initially with contacts known to the authors, the wider research team and the funding body. A horse welfare assessment information sheet stating the purpose of the research, method of data collection, participant’s rights, data storage and usage, time commitment, confidentiality/anonymity and the absence of economic benefits for participating in the study was given/read to participants. A consent form was used for horse owners to give initialled consent to the assessment of their horse/horses. A protocol setting out the measures to be taken if the researcher and/or wider research team witnessed the mistreatment of animals, or a disclosure is made of animal neglect and/or abuse was devised.

From the horse owners who consented for their horses to be assessed, a convenience sample of 104 horses was obtained. Horses were assessed at different locations throughout the UK and Ireland: Appleby Horse Fair, (Cumbria, England), Ballinasloe Horse Fair (Ireland), BHS horse health clinics, and horse owners’ homes and yards. Data were collected between May 2018 and June 2019. For health and safety reasons and to ensure standardisation across multiple different contexts, all horses were assessed visually from a distance of 1.5 metres. The welfare assessment took approximately ten to fifteen minutes per horse to complete, and ambient environmental temperature was also recorded, using the BBC Weather app.

The assessments started with QBA and observations lasted from two to three minutes. The individual horse was scored by placing a mark on the visual analogue scale that best reflected the strength of the horse’s expression for each of the QBA terms. Scores were recorded on the tablet, and data were transferred to a common server at the end of each day for storage. Horses were then assessed according to the welfare assessment protocol outlined above.

### 2.5. Statistical Analysis

Data were initially cleaned and entered into IBM SPSS Statistics 25 (Chicago, IL, USA) for statistical analyses. Unless stated otherwise, all animals were included in analysis. Horse breeds were regrouped into the following categories: Cobs, Trotters and ‘Other’ (Connemara, Shetland, Dales and New Forest ponies, and crossbreeds). Descriptive statistics were conducted on all questions to provide a general view of the data. Results of welfare assessments of horses in their home environment reflected those of horses assessed in fairs/clinics, therefore, it was deemed appropriate to pool the data for analysis.

Using logistic regression, models were built to determine the effects of independent variables on various dependent variables. The models were built by selecting and entering predictors which were considered relevant to the dependent variables, e.g., the relationship between poor body condition and body lesions. Screening of variables was used to remove the variables that did not contribute to the model. This practice of deleting and reinstating variables continued until variables that remained in the model contributed to a model that was a good fit to the data (Hosmer—Lemeshow χ^2^ test). Variables were coded as 0 = absent and 1 = present. Tests to identify whether the assumption of collinearity was met revealed that multicollinearity was not a concern. Principal Component Analysis (PCA–correlation matrix, no rotation) was used to assess the correlation between the 14 QBA descriptors and to summarise the data into principal components. Coefficients with a loading of at least 0.30 were chosen for factor analysis [33]. Preliminary assessments of factors were based on eigenvalues, cumulative variance and visual examination of the scree plot and factor loadings.

To assess the relationship between QBA and other horse welfare measures collected for this study, a new file was created in SPSS 25, with both PCA dimension and horse welfare scores. A general linear model (GLM) was used to assess the relationship between QBA, and horse welfare measures and the residuals were checked for normality. Post hoc analysis (Tukey test) was used to determine where differences lay. A median BCS for all three body areas was created for the purposes of analysis. R studio 1.2.5019 (with package FactoMinerR) was used to create QBA bi-plot graphs. A significance level of *p* < 0.05 was used, and significant values are in bold.

## 3. Results

Horse Information: In total, 104 horses were assessed for this study. The majority (58.7%, *n* = 61) of horses assessed were Cobs, 24% (*n* = 25) were Trotters and 17.3% (*n* = 18) were other breeds of horses. Horse ages ranged in age from 6 months to 23 years ($\bar{x}$ = 5.5, σ = 4.2). There were 56 (53.8%) mares, 24 (23.1%) stallions and 24 (23.1%) geldings. Of the 41 mares where data were collected, five (4.8%) had foals at foot and the remainder (36, 34.6%) were not in foal. Of the horses, 55 (52.8%) were tethered, 11 (10.5%) were loose in a field, 3 (2.8%) were stabled individually, 1 (0.96%) was stabled in a group and 34 (32.6%) were assessed in a different situation which included being hand-held, attached to a gig and/or standing in a yard.

Of the 104 horses assessed, 46 (44.2%) were assessed at Appleby Horse Fair and 19 (18.2%) at Ballinasloe Horse Fair. At horse health clinics, 12 (11.5%) horses were assessed in Wales and 6 (5.7%) in northeast England. In their home environment, 16 (15.3%) horses were assessed in Ireland and 5 (4.8%) in Scotland. In total, 44 (42.3%) horses were assessed in the summer, 38 (36.5%) in spring, 19 (18.3%) in autumn and 3 (2.9%) in winter. Ambient temperatures ranged from summer, 10°–25 °C ($\bar{x}$ = 17.42 σ = 4.33), spring, 8.5°–18 °C ($\bar{x}$ = 11.10 σ = 2.30), autumn, 8°–10 °C ($\bar{x}$ = 9.31 σ = 0.82) and winter, 7 °C.

### 3.1. Horse Welfare Assessments

**Figure 1:**
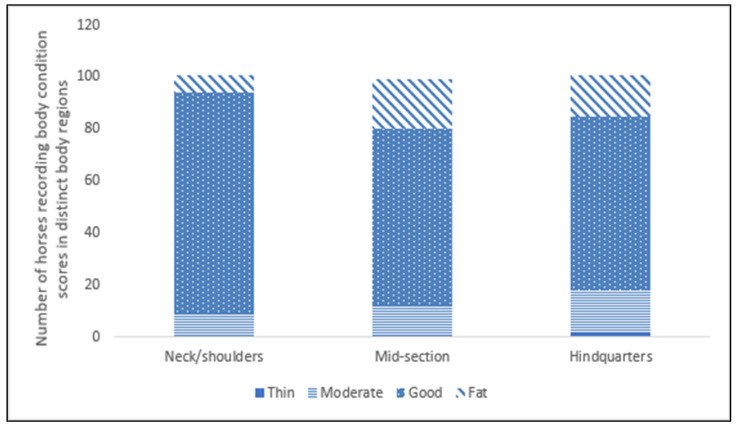
Number of horses (*n* = 101) recording body condition scores on distinct body regions (Scale 0 = very thin, 1 = thin, 2 = moderate, 3 = good, 4 = fat, 5 = very fat, Carroll and Huntington, [27]).

Body condition score and cresty neck: An overall body condition median score of 3 (Good) was recorded for 81% (*n* = 84) of the 104 horses assessed. Of the remaining horses, 9.6% (*n* = 10) were recorded as fat (Score 4), 1.9% (*n* = 2) had poor body condition (Score 1) and 7.7% (*n* = 8) were moderate (Score 2). Three horses were assessed in the winter and were found to have an acceptable overall BCS as adapted for winter coat. BCS for each body section indicated that the majority of horses obtained a score of 3 (Good) (Figure 1). Of the 104 horses, 99 (95.2%) had no visible crest and 5 (4.8%) had a slight visible crest.

Hair coat condition: The majority (76%, *n* = 79) of horses had a healthy/sleek and glossy hair coat condition while only around 8% (*n* = 8) had an unhealthy/dry/dull coat. A muddy coat from negative environmental conditions was assessed on 8.7% (*n* = 9) of horses, and 7.7% (*n* = 8) of horses were assessed with a muddy coat from positive activities such as rolling.

### 3.2. Skin Conditions

Body Lesions: Body lesions were absent from the majority (77.9%, *n* = 81) of horses. There were no serious lesions recorded. Of the body lesions that were recorded, minor injury/cuts through the skin on the muzzle and neck and superficial/healed lesions were the most common types of lesions.

Hairless Patches: Hairless patches were absent on the majority of horses (83.7%, *n* = 87), if present, the midsection had the most hairless patches (2.9%, *n* = 3).

Sunburn/Rain Scald: The majority (96.2%, *n* = 100) of horses did not have sunburn and rain scald was not recorded on any of the horses assessed.

Skin Irritation on Lower Legs/Pastern: There was no evidence of mud rash and dermatophilosis on the horses assessed (62.9%, *n* = 66). Three (2.9%) horses, showed signs of skin irritation on one leg.

**Figure 2:**
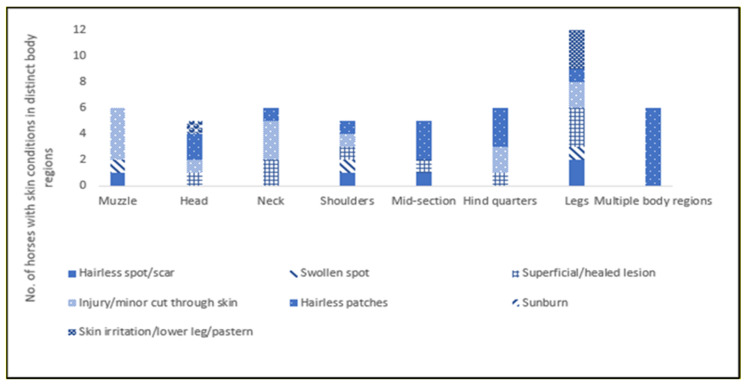
Number of horses with different types of skin conditions (body lesions, hairless patches, sunburn and skin Irritation 0n lower legs/pastern) recorded in distinct body regions.

A total of 29 (27.8%) horses had body lesions (hairless spot/scar, swollen spot, superficial/healed lesion, injury/minor cut through skin) on different body regions. Of these, lesions on the legs were the most prevalent (5.8%, *n* = 8). Six horses had hairless patches in multiple body regions (Figure 2).

**Figure 3:**
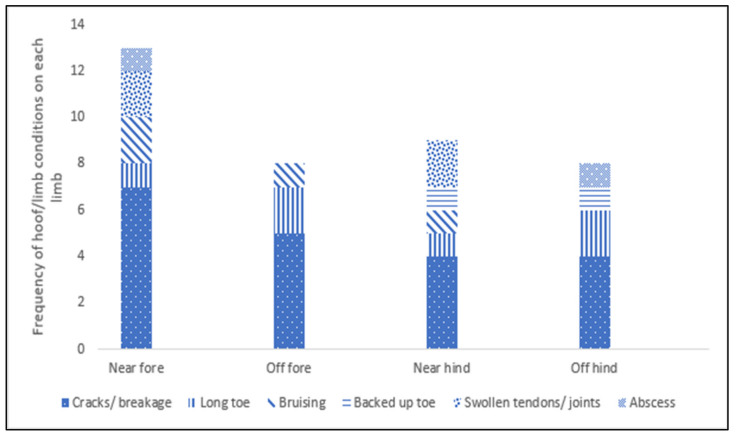
Frequency of hoof/limb conditions on each limb of horses assessed.

Limb/Hoof conditions: Five (4.8%) horses had swollen tendons/joints; one (1%) in the elbow, three (2.9%) in the carpus and one (1%) in the fetlock of the near fore and near hind limbs. Just over half (51.9%, *n* = 54) of horses were not shod, 47.1% (*n* = 50) were shod on all four feet and one horse (0.96%) was shod, but two of the shoes had fallen off. There was no evidence of hoof neglect in 73.1% (*n* = 76) of horses while 26.9% (*n* = 28) of horses displayed signs of hoof neglect. Hoof wall cracks/breakages and long toes were the most common hoof conditions recorded. Cracks/breakages, long toe and bruises were evident on all four hooves (Figure 3). Of the 83 horses assessed for growth rings, the majority of horses did not display prominent growth rings (76.9%, *n* = 80).

Heat stress: Of the 83 (79.8%) horses assessed for heat stress, 6 (5.8%) horses displayed sweating, 3 (3.6%) flared nostrils, 2 (1.9%) increased respiration rate and 2 (1.9%) apathy. None of the horses displayed four of the criteria that indicated heat stress (Table 2). *Ocular/nasal discharge/cough*: Occular discharge was absent in 98.1% (*n* = 102) of horses, and nasal discharge was absent from most horses (93.3%, *n* = 97). Further, 96.2% (*n* = 100) of horses did not cough in a 5-min period during assessment.

### 3.3. Horse Management

Shelter*:* Shelter was available for 67 (64.4%) horses, while shelter was unavailable for 37 (35.6%) horses. Ninety-eight (94.2%) horses had access to a dry, mud free standing area while six (5.8%) did not.

Water availability: Water was available for 79 (76%) horses, unavailable for 18 (17.3) horses and evidence of water availability was seen for 7 (6.7%) of horses. For those 79 horses where water was available, 77 (74%) had access to a single water point while 2 horses (1.9%) had access to 2 distinct water points.

### 3.4. Horse Behaviour

Abnormal Behaviours (e.g., crib biting, wood sucking/chewing, weaving): Only one (0.96%) horse displayed any evidence of abnormal behaviours (wood chewing).

Voluntary animal approach test: When horses were assessed for voluntary animal approach, most (80.8%, *n* = 84) demonstrated a friendly response, 18 (17.3%) a negative reactive response and 2 (1.9%) a negative non-reactive response.

### 3.5. The Effects of Horse Welfare Characteristics and Horse Management Practices on Horse Welfare Indicators

An ordinal logistic regression determined the relationship between horse characteristics, other welfare assessment parameters and horse body condition. The deviance goodness-of-fit test indicated that the model was a good fit to the observed data, χ^2^ (159) = 89.59, *p* = 0.56. The final model significantly predicted the dependent variable over and above the intercept-only model, χ^2^ (12) = 28.13, *p* < 0.01. Four of the twelve predictor variables were found to be statistically significant. Horses were more likely to have good body condition (Score 3) when skin irritation (lower leg/pastern) (*p* < 0.01) and signs of hoof neglect were absent (*p* < 0.01*)*. Good body condition (Score 3) was less likely when horses were <4 years old, rather than 4–15 years old (*p* = 0.03), and when generalised skin conditions were present (*p* = 0.01) (Table 3).

A binomial logistic regression determined the relationship between horse characteristics, other welfare assessment parameters and the presence of hoof neglect. The deviance goodness-of-fit test indicated that the model was a good fit to the observed data, χ^2^(8) = 7.39, *p* = 0.49. The binomial logistic regression model was statistically significant, χ^2^(13) = 38.86, *p* < 0.01, explained 47% (Nagelkerke R^2^) of the variance in the presence of hoof neglect and correctly classified 82% of cases. Four of the twelve predictor variables were statistically significant. The presence of hoof neglect was significantly more likely when poor coat condition (*p* < 0.01) and body lesions (*p* = 0.03) were present and when horses did not show friendly responses to the approach test (*p* < 0.01). Hoof neglect was less likely as body condition score increased (*p* = 0.02) (Table 4).

A binomial logistic regression determined the relationship between horse characteristics, other welfare assessment parameters and the presence of hoof cracks/breakages. The deviance goodness-of-fit test indicated that the model was a good fit to the observed data, χ^2^(8) = 10.9, *p* = 0.20. The model was statistically significant, χ^2^(13) = 26.16, *p* < 0.01, explained 31% (Nagelkerke R^2^) of the variance in the presence of hoof cracks/breakages and correctly classified 70% of cases. Three of the thirteen predictor variables were statistically significant. The presence of hoof cracks/breakages was significantly more likely when hairless patches (*p* = 0.04) and body lesions (*p* < 0.01) were present and tended to be less likely when skin irritation (lower leg pastern) was present (*p* = 0.06). There was a tendency for hoof/cracks/breakages to be more likely when poor coat condition was present (*p* = 0.07) (Table 5).

A binomial logistic regression determined the relationship between horse characteristics and welfare assessment parameters on the likelihood of tethering horses. The deviance goodness-of-fit test indicated that the model was a good fit to the observed data, χ^2^(8) = 4.17, *p* = 0.84. The logistic regression model was statistically significant, χ^2^(5) = 24.59, *p* < 0.01, explained 28% (Nagelkerke R^2^) of the variance in horse tethering and correctly classified 72% of cases. One of the six predictor variables was statistically significant. Tethering of horses was more likely when shelter was present (*p* < 0.01) (Table 6).

A binomial logistic regression determined the relationship between horse characteristics, other welfare assessment parameters and the response to the voluntary animal approach test. The deviance goodness-of-fit test indicated that the model was a good fit to the observed data, χ^2^(8) = 8.24, *p* = 0.41. The model was statistically significant, χ^2^(16) = 29.98, *p* < 0.01, explained 40% (Nagelkerke R^2^) of the variance in the voluntary animal approach test and correctly classified 85% of cases. Of the fifteen predictor variables, only one was statistically significant. A friendly response to the voluntary animal approach test was more likely when signs of hoof neglect were absent (*p* = 0.02), and there was a tendency for a friendly response to be more likely from Trotters than other breeds (*p* = 0.07) (Table 7).

### 3.6. Qualitative Behaviour Assessment (QBA)

QBA was conducted on 84 horses from the main dataset (*n* = 104). The QBA ratings generated scores for each horse on the 14 behavioural expression descriptors. Descriptive statistics indicated a relatively high mean score for ‘friendly’ ($\bar{x}$ = 67.10 σ = 37.50), ‘good form’ ($\bar{x}$ = 55.54 σ = 37.45), and ‘settled’ ($\bar{x}$ = 53.59 σ = 38.41) and a lower mean score for ‘pushy’ ($\bar{x}$ = 10.46 σ = 15.51), ‘down’ ($\bar{x}$ = 8.10 σ = 15.34) and ‘aggressive’ ($\bar{x}$ = 4.98 σ = 3.00). PCA detected two main principal components (PC1 mood. PC2, energy) with eigenvalues greater than 1, together explaining 56.9% of the total variance (Table 8).

**Figure 4:**
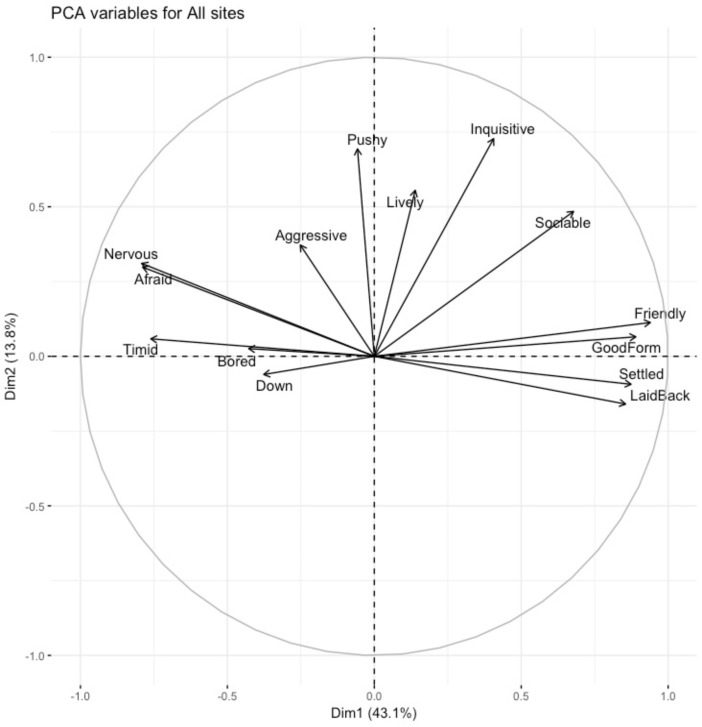
Bi-plot of descriptor loadings on Dimension 1 (mood) and Dimension 2 (energy).

The distribution of descriptors for the two PCA dimensions are shown in a two-dimensional loading plot. PC1 (10 items) explained 43.1% of the variance and ranged from ‘friendly/good form’ to ‘afraid/nervous’ which contrasts between positive and negative mood. This dimension was labelled ‘mood’. PC2 (4 items) explained 13.8% of the variance and describes how inquisitive/pushy the horses were. This dimension was labelled ‘energy’ (Figure 4).

### 3.7. The Relationship between QBA Scores and Welfare Assessment Parameters

**Figure 5:**
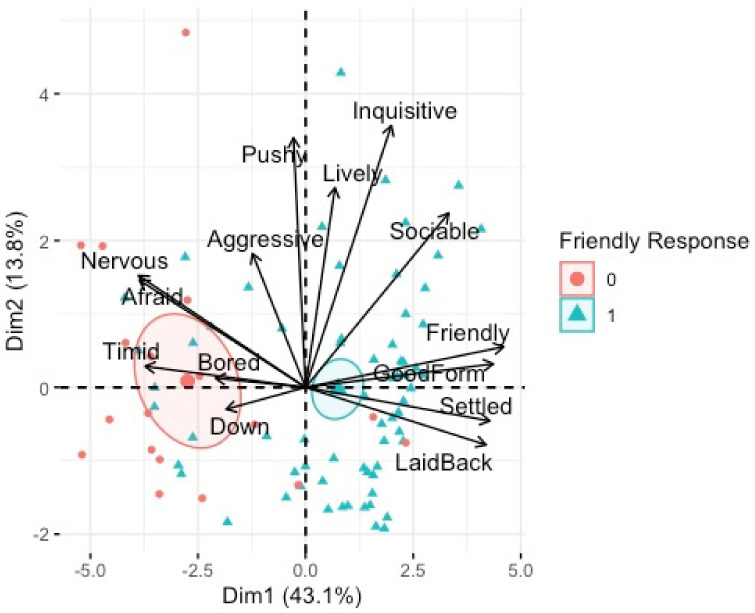
PCA bi-plot of QBA terms associated with individual horses who displayed a friendly response to the voluntary animal approach test (1) or not (0). Dimension 1 (mood) and Dimension 2 (energy).

**Figure 6:**
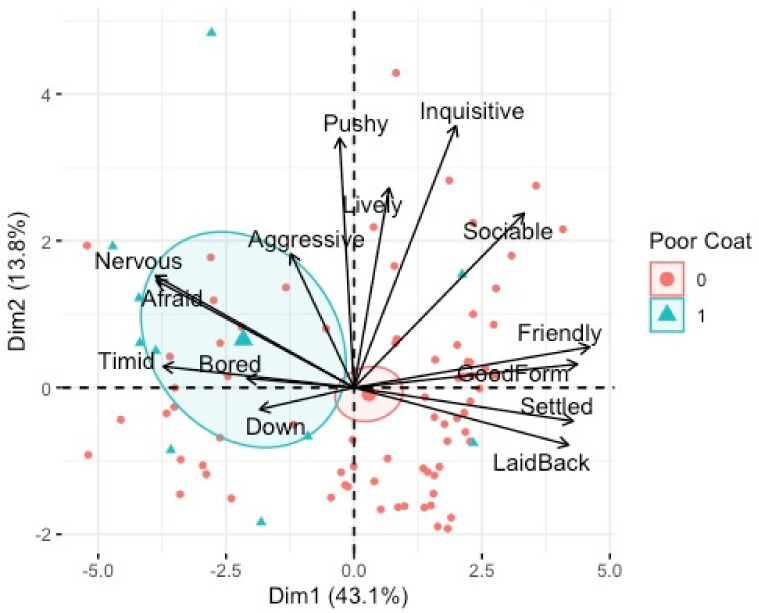
PCA bi-plot of QBA terms associated with individual horses who had a poor coat condition (1) or not (0). Dimension 1 (mood) and Dimension 2 (energy).

Data from 84 horses were used to examine the relationship between QBA and the overall horse welfare measures. Both PC1 (mood) and PC2 (energy) met the assumptions of normality. Results of a GLM indicated that there was a statistically significant relationship between PC1 (mood) and a friendly response to the voluntary animal approach test, F(_1, 78_) 4.96*, p* < 0.01*,* adj. R^2^ = 0.37, and there was a tendency for a significant relationship between PC1 (mood) and poor coat condition, F(_1, 78_) –1.84*, p* = 0.06, adj. R^2^ = 0.37. Post−hoc analyses revealed that horses that showed a friendly response to the voluntary animal approach test had significantly higher PC1 (mood) (Figure 5). There was a tendency for horses with poor coat condition to have lower PC1 (mood) (Figure 6).

**Figure 7:**
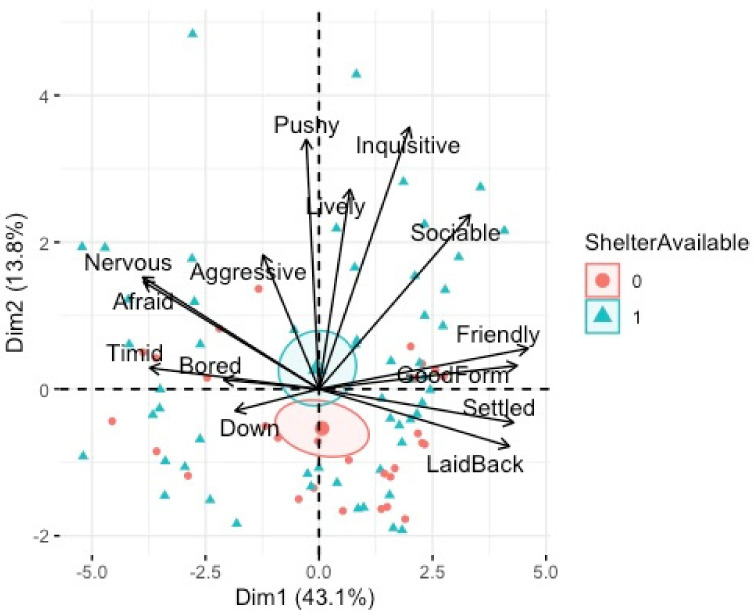
PCA bi-plot of QBA terms associated with individual horses who had shelter available (1) or not (0). Dimension 1 (mood) and Dimension 2 (energy).

**Figure 8:**
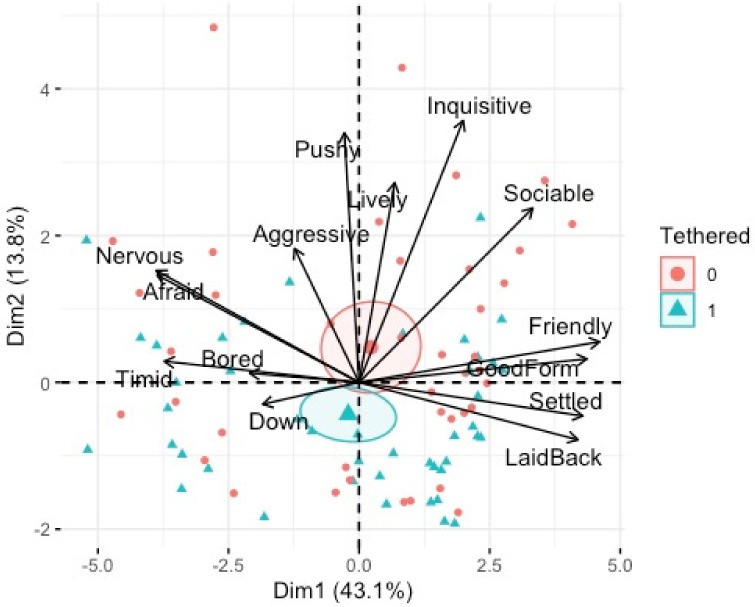
PCA bi-plot of QBA terms associated with individual horses who were tethered (1) or not (0). Dimension 1 (mood) and Dimension 2 (energy).

**Figure 9:**
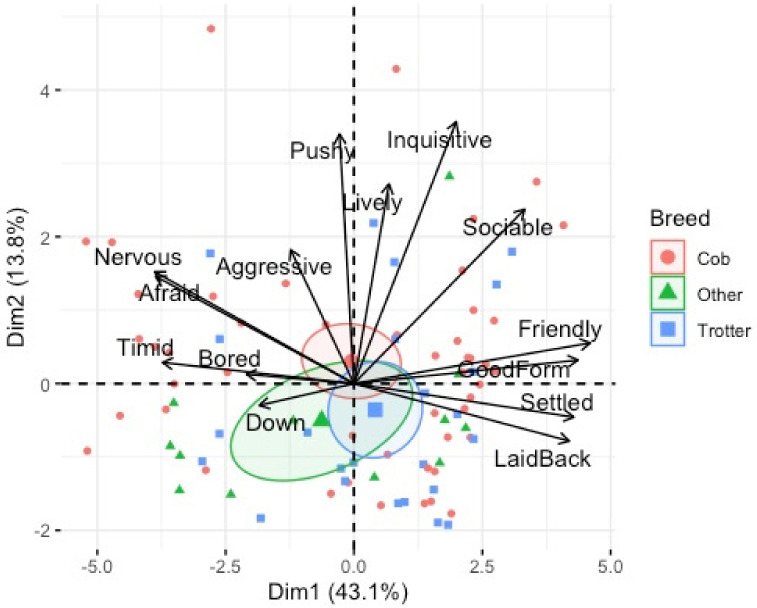
PCA bi-plot of QBA of terms associated with individual horses who were either a Cob, Trotter or other. Dimension 1 (mood) and Dimension 2 (energy).

There was a statistically significant relationship between PC2 (inquisitive/pushy) and shelter availability, F(_1, 75_) 5.70, *p* < 0.01, adj. R^2^ = 0.26, tethering, F(_1, 75_) 3.42, *p* < 0.01, adj. R^2^ = 0.26, poor coat condition F(1, 75) 3.60*, p* = 0.04, adj. R^2^ = 0.26 and breed of horse, F(_2, 75_) 6.29*, p* = 0.02, adj. R^2^ = 0.26. Post-hoc analyses revealed that horses that had shelter available had significantly higher PC2 (energy) (Figure 7). Horses with poor coat condition had significantly higher PC2 (energy) (Figure 6). Tethered horses had significantly lower PC2 (energy) (Figure 8), and Cobs had significantly higher PC2 (energy) than Trotters and other breeds (Figure 9).

## 4. Discussion

### 4.1. Prevalence of Welfare Issues

Horse body condition: In the present study, most Traveller and Gypsy owned horses had a ‘good’ (3) BCS score or an adequate winter BCS, and most had no visible cresty neck. Further, an optimal score was recorded on most horse regional body areas. These results perhaps indicate that Traveller and Gypsy horse owners are managing their horses in a way that is conducive to a ‘good’ body condition, and this concurs with previous findings where Traveller horse owners identified BCS as a key factor in horse health care [34]. A very small percentage of horses in the present study had a suboptimal BCS, although there were no horses at either of the extremes (very poor or very fat) of the scale and only two horses with ‘poor’ body condition. There are several reasons for a lower than optimal BCS, such as parasitism, malnutrition, poor dentition, etc. [35], or it may be that lower BCS scores in this study may be related to seasonal variation in body condition. Over a third of horses were assessed during the late winter and spring months, and previous studies have suggested that horse BCS varied between seasons, with scores greater in the spring and summer and lower in the winter [36]. Such seasonal adaptations provide for winter forage shortages [37,38]. As Traveller and Gypsy owned horses are usually kept outdoors and generally not managed as intensively as leisure horses, [34], it is likely that they undergo some natural seasonal variation in body condition. Further, the BCS system in this study was based on visual assessment alone, therefore, scores may not be considered as accurate as those assessed using a conventional BCS assessment, where palpation is used to determine body condition. In addition, Cobs have thick coats, and Cobs were the principal breed of horse assessed in this study; therefore, visual BCS through the hair coat may also have impacted scores, as coat thickness was a characteristic found to reduce the accuracy of BCS [39]. However, most horses in this study were assessed during the spring and summer months when they were less likely to possess a long or thick coat.

In this study, an absence of hoof neglect was associated with good body condition, which agrees with findings from previous studies which found that a lower body condition was associated with the presence of hoof neglect [40,41], and it may be that a low body condition score is indicative of general neglect and lack of overall care [42]. However, it was also found that good body condition was more likely in the presence of generalised skin conditions. This result may be explained by conditions such as sweet itch, which is an allergy rather than directly related to management. Further, as it is caused by an allergy to midges (*Ceratopogonidae),* it is prevalent at the time and in the places where it is warm and damp, conditions which are also optimal for grass growth and, therefore, increased BCS in horses. Younger horses (<4) were less likely to have good body condition than horses over 20 years, which may be a result of younger horses still growing or perhaps having less access to resources due to the social organization within a herd [43]. Young horses may also be more susceptible to gastrointestinal parasites, which may impact their body weight, with infections such as roundworms (*Ascarids*) and threadworms (*Strongyloides*) more commonly found in this age group [44]. However, horse body condition for the majority of Traveller and Gypsy owned horses in this study was good which is in contrast with the findings from other studies of body condition in leisure horses. Equine obesity is a growing welfare issue, with 72% of ponies aged ≥7 years found to be overweight or obese in a study conducted within a 50-mile radius of The Royal Veterinary College in England [45].

Hair/coat condition: Most horses had a healthy hair coat or a healthy muddy coat, indicative of rolling. Good hair coat condition is often seen as a reflection of a horse’s health [29]. In a previous study, Traveller horse owners referred to hair coat condition as an indicator of their horse’s health status [34]. Nutritional deficiencies, presence of parasites, skin infections, internal diseases or poor husbandry conditions may all affect hair coat condition [46,47]. Therefore, the use of hair coat condition as a welfare indicator can be very advantageous in that it may help horse owners identify initial health concerns. However, a small percentage of horses had an unhealthy or muddy coat associated with being kept in muddy, wet conditions. There was a relationship between poor coat condition and lower mood and higher energy. Previously, Lesimple suggested that hair coat condition is associated with positive mood [48]. This may be due to the link between health, coat condition and mood, but may also be associated with the previous point regarding horses being kept in wet, muddy conditions, which may also impact their mood. However, further investigation is required, as this finding was only a tendency rather than statistically significant. A relationship between energy and poor coat was also found, with higher energy being associated with poor coat condition. This may be a sign of a lack of general handling, which may mean that these animals cannot receive basic care such as grooming or veterinary. Previous studies have indicated that the welfare of horses that are difficult to handle may be compromised [49].

Body lesions: There were few lesions recorded in this study and none were serious. Luna et al. also found that lesions on Chilean horses were simple abrasions and were largely in areas associated with harnessing, although, in their study, horses were work rather than leisure animals [50]. Nevertheless, the welfare state of the two horse-owning communities is often reported to be poor [11,51]. In the present study, the lesions documented were an injury/minor cut through the skin and superficial/healed lesions, which indicates old injuries, with most located on the legs. As most horses were assessed at horse fairs or health clinics, it is possible that injury/minor cuts may have been sustained during transportation. This finding is supported by Riley et al., who reported 25% horse transportation related injuries in their study on transporting horses to equestrian events in Australia [52].

Skin conditions: A minority of horses had hairless patches and skin irritations. Skin disease was the most common disease syndrome recorded in the National Equine Health Survey [53], although, in the present study, it does not appear to be a common complaint in Traveller and Gypsy owned horses. This may be associated with the predominant breed of horses assessed. Cobs have thick coats; therefore, these conditions may be less visible to the observer, particularly as the assessment did not allow for palpation for detection of skin conditions hidden below the hair. However, it may also be that a healthy thick hair coat may provide protection from insects and micro-organisms and help protect the legs from moisture and mud and, therefore, mud fever *(Dermatophilosis, pastern dermatitis).* Further, horses kept outdoors are less likely to have their legs washed than horses kept in stables; therefore, they are not exposed to the frequent wetting, scrubbing and drying of the skin, which has been indicated as potentially exacerbating mud fever. However, to the researcher’s knowledge, there are no empirical studies to support this viewpoint. Further, the low levels of rugging and the high levels of turnout, as found in this study, are likely to lead to healthier skin and coats [54,55].

Discharges: Ocular and nasal discharge and cough were uncommon in assessed horses, and, of those who presented with these issues, clear watery discharge was the most prevalent. Clear, watery discharge is not usually a concern if the horse is otherwise in good health [56], although identification of the origin and nature of any discharges and coughs would help rule out any significant concerns. This is particularly important in the context of unregulated horse fairs, where horse health and welfare may be compromised due to the large number of horses from different regions and countries in attendance. Stakeholders identified unregulated horse fairs as an area of key concern for horse welfare [13]. While not assessed in horses in this study, infectious respiratory diseases such as *Streptococcus equi var. equi* infection (Strangles) and equine influenza can increase the risk of serious or life-threatening complications [57,58]. These are highly contagious diseases and have the potential to spread at horse fairs. It must be noted that at all other equine events, a passport with up-to-date vaccinations is required for participation, to counter disease outbreaks. Nevertheless, Traveller and Gypsy owned horses in this study displayed few, if any, clinical signs of these health conditions.

Hoof condition: Hoof neglect and the presence of hoof conditions was the most frequently recorded welfare issue. Nearly a third of horses presented with hoof neglect, and common hoof conditions recorded were hoof wall cracks/breakages and long toe, with both conditions evident in all four limbs. These conditions may be attributed to owners’ lack of knowledge of hoof care in general. With over half of the horses in this study unshod, owners may not be aware of the need for regular hoof care in barefoot horses. However, horse hoof conditions are widely reported [51,59,60] and were one of the most common welfare issues assessed in horses sent to slaughter in the U.S. [61]. Further, the effect of season and environment on horse foot health has been documented. For example, Ley et al. found that seasonal trends considerably affected hoof wall strength in Thoroughbred mares [62].

The presence of hoof neglect in this study was significantly more likely when poor coat condition and generalised skin conditions were present and when horses did not show friendly responses to the approach test. Again, this likely shows a general lack of handling and, possibly, neglect. In addition, if horses are difficult to handle/catch, as shown by the approach test, then it will be difficult to treat or manage any conditions. Further, hoof neglect was less likely as BCS increased. These findings are consistent with those of Fröhlich et al., who found that hoof neglect was associated with low body condition scores in working horses in Fiji and was thought to be associated with general lack of care and neglect [41]. It must be noted that the likelihood of hoof neglect was not associated with the highest BCS (very fat), as this was not recorded in horses in this study. Obesity in horses increases the risk of laminitis [63]. Chronic laminitis can result in very long underrun and deformed hooves, which need to be treated with regular care and a strict management routine [64].

While the hoof conditions presented in the current study were relatively mild, there was significant evidence of hoof neglect which, while not unique to Traveller and Gypsy owned horses, does require further investigation and possible further targeted interventions.

### 4.2. Horse Management

Shelter: In all assessment environments, most horses had access to shelter and/or a dry mud-free standing area. Shelters ranged from purpose-built stables to natural shelter such as hedges and trees, with most horses provided with the latter. It is common for Traveller and Gypsy owned horses to live outdoors in naturalistic conditions [34], therefore, the observation that natural shelter was more common than purpose-built shelter was perhaps not surprising. Jørgensen et al. observed, in their study on Nordic horses’ preference for shelter, that most horses remained outdoors, and, in addition to a thick coat, a dry lying space was found to improve the horse’s capacity to acclimatise to low temperatures [65,66]. However, outdoor horses were found to use shelter areas more during times of precipitation, low temperatures and strong winds [67,68,69], therefore, access to shelter is necessary for improved horse welfare. A possible explanation for lack of shelter for some horses in this study may be that these horses were assessed away from their normal living areas, therefore, it is probable that shelter may be available outside of the assessment time. In addition, horses that had shelter had higher energy (inquisitive/pushy). This could be due to a horse’s greater confidence while in their home environment and while with their friends versus those at a fair or clinic, who are away from home and possibly isolated. However, no statistical relationship was found between being assessed in the home environment, shelter availability and energy levels, so this is a proposition which requires further investigation.

Tethering*:* Over 50% of horses in the present study were tethered, although the tethering of horses was predominately observed in fair situations. However, some horses were also tethered in their home environments. Previous studies have found that access to land is a major barrier to keeping horses for this group, which means that they often tether [4,34]. Tethering horses is not considered to be good horse management [70]. Nevertheless, tethered horses had fewer physical welfare issues and were less likely to be thin than roaming horses in a study on the welfare of long-line tethered and free-ranging horses in South Wales [32]. In fact, the tethering of horses in this study was more likely when shelter was present, indicating that horse owners were aware of the need for the horse to have access to shelter when required and when they are unable to seek it themselves due to being tethered. Findings from QBA assessment revealed that tethered horses had lower energy (inquisitive/pushy). As tethering is a practice that restricts a horse’s movement, the opportunity to exhibit high energy behaviours is limited, and a previous study found that grazing, a low energy activity, was found to account for most of tethered horses’ time-budget [32]. For the safety of the horse, it is important that horses are calm whilst tethered, therefore, there is likely to be a period of training and acclimation to the practice of tethering. Further, it may be that tethered horses are more likely to remain calm, as they are surrounded by other horses (rather than isolated) and have access to a visual horizon which provides them with a view of conspecifics. Increased visual contact among stabled horses was found to decrease the risk of stereotypic behaviours [71].

Water availability: Water from a single water point was available for most horses. The presence of water is an important welfare indicator [72]. In fact, the absence of prolonged thirst has been identified as an iceberg indicator in the assessment of horse welfare [29]. An iceberg indicator tends to be related to the state of other welfare measures and is used to indicate welfare issues at the animal level [73]. Therefore, it is positive to observe that three quarters of Traveller and Gypsy horse owners provide water to their horses. This finding is contrary to that of Mullan et al., who found that tethered horses had limited access to water [32]. Their study differs from the present study, as horses were assessed in a single location on a Welsh Common while, in this study, horses were assessed in various situations throughout the UK and Ireland, which may be considered as a more representative sample.

### 4.3. Horse Behaviour

Abnormal behaviours: Abnormal behaviours such as stereotypies were absent from all but one horse. One of the main effects of domestication for horses is the limitation for horses to freely exhibit natural behaviours such as foraging and social contact. Consequently, stereotypies which are commonly based on feeding and locomotory behaviours are likely to develop [74]. Therefore, the absence of stereotypies in this study may indicate that Traveller and Gypsy horse owners provide their horses with environments that are more comparable to their natural conditions. This finding is supported by Rowland et al., who found that Traveller horse owners had a relatively good understanding of the horses’ natural behaviour and their environment, and this was reflected in their management practices [34].

Voluntary animal approach: Most horses demonstrated a positive (friendly response) in the voluntary animal approach test. This test gives an indication of how the animal is interacting with its environment and its owners/handlers [35,75]. Therefore, the high incidence of a friendly response towards the researcher in this study supports the notion that a positive human−animal relationship between Traveller and Gypsy horse owners and their horses exists. The relationship between mood (friendly/good form) and the presence of a friendly response to the voluntary animal approach test was to be expected. Horses in a positive emotional state are more likely to approach humans in a friendly way. This result is supported by Minero et al., who stated that the horse’s self-confidence and its welfare is influenced by the human−horse relationship [49].

However, in this study, some horses did exhibit negative reactive responses. For horses that responded in a negative reactive way, reasons recorded included lots of activity in the area of assessment, first time at the fair, tethered with other horses and awaiting medical procedure. While these are stressful events and may have contributed to the negative response, adverse reactions may also be an indication of a poor human−horse relationship [76].

QBA: Positive QBA terms exceeded negative terms. This finding indicates that the emotional states of Traveller and Gypsy owned horses were characterised by more positive affective states, perhaps refuting the concern for the welfare of these horses [11,13]. In addition, the two main QBA dimensions identified both positive and negative emotional connotations. The first dimension represented the terms good form/friendly versus afraid/nervous, which reflects different aspects of mood, and the terms in the second dimension ranged from inquisitive/lively versus aggressive/pushy, reflecting aspects of energy. This dimensionality is supported by QBA studies in pigs [77], goats [78] and donkeys [22]. It is interesting that most of the terms loaded highly on the first dimension (mood), with these terms going between friendly/good form and nervous/afraid, and it may be that the atmosphere at fairs/clinics heightened both positive and negative mood.

Limitations: The use of convenience sampling may have contributed to potential bias; therefore, results cannot be generalised. However, assessments took place in a number of venues and locations throughout the UK and Ireland. Further, Traveller and Gypsy owned horses were only assessed on one occasion; therefore, research with a larger sample size and potentially also repeated assessments, are required to show the generalisability of results.

Most study sites where assessments took place were not the horses’ normal home environment; consequently, the findings may not be representative of normal horse owner management practices and horse behaviour. Therefore, it is recommended that additional horse welfare assessments are conducted in the horse’s home environment, as well, to enable a more comprehensive and representative assessment of welfare. The fixed list of QBA terms was found to be useful as a quick, easy-to-use and non−invasive tool to measure horse behavioural expression in this study. However, it must be noted that QBA was carried out by the main researcher alone, who had a good knowledge of horse behaviour and intensive training in QBA. While these qualities are required for reliable and valid scoring [19], the reliability and validity of this tool for Traveller and Gypsy owned horses needs further investigation. Testing of QBA scoring of the same assessor at different points of time and/or scoring between different assessors will determine the reliability of QBA terms used in this study.

## 5. Conclusions

The aims and objectives of this study were to identify and describe the welfare status of Traveller and Gypsy owned horses in the UK and Ireland. The development of a horse welfare assessment tool for this study was valuable in identifying potential welfare concerns in these horses. The overall assessment indicated that horse health and welfare was of a good standard, with an optimal body condition score and the absence of skin conditions observed in most horses. However, hoof neglect and the presence of hoof conditions was evident in about a quarter of horses assessed. The results of the QBA study captured many positive elements of horse expressive behaviours. While Traveller and Gypsy horse owners are often referred to as ‘hard to reach’, the current study was successful in generating a list of QBA terms in consultation with this group, indicating that they are invested in their horses’ welfare.

This study is the first of its type to begin to quantify the welfare of Traveller and Gypsy owned horses. Although further research is required to ensure generalisability to all horses within this population, these results are a good starting point on which to engage with stakeholders who previously identified Traveller and Gypsy owned horses in the UK and Ireland to be particularly vulnerable to poor welfare. In addition, these data will allow the development of targeted interventions, based on the findings, to address those areas where horse welfare was considered to be suboptimal.

## Figures and Tables

**Table 1:** List of QBA descriptors and agreed synonyms and explanations from the Traveller focus group.

List of QBA Descriptors	Agreed Synonyms/Explanations
Nervous	shakes, muscles are tense, tail is tucked under them
Timid	shy, stays in the background
Sociable	mixes well with other horses
Settled	relaxed on their own and with other horses, content
Afraid	anxious, sweating, shivering, rears up, avoids people
Good form	happy, friendly, lively, rolling, bucking, kicking
Laid back	relaxed, easy going, gets on with their own thing
Inquisitive	nosy, curious, interested in everything
Lively	full of energy, cheerful, running about
Down	depressed, by themselves, head down, no interest in anything, might be off their food
Aggressive	biting, kicking, snarling, tail swishing, would turn towards you or other horses
Friendly	warm, easy to approach, will come up to you
Bored	not interested in anything, just standing there, needs something to do
Pushy	demanding, pushes everything out of their way to get what they want

**Table 2:** Welfare assessment protocol, including indicators, criteria of use, method of use and classification.

Indicator	Criteria	Method	Classification
Demographic descriptors	Descriptors: date of assessment, age, breed, sex, season, ambient environmental temperature, geographical region/location	Record descriptors for each horse	Date, Age, Breed, Sex, Season, Ambient Temperature, Region and Location
Health measures			
Body Condition *Adapted from Carroll* *and Huntington’s* *system, [27]* *Adaptation for Winter Coat (created for this study)*	Assessed using an averaging scoring system with the BCS system as follows: Section 1*: Neck and Shoulder (withers is cut-off point)* Section 2*: Middle -Back* *and Belly (last rib* *is cut-off point)* Section 3*: Bottom–Pelvis to Tail* A winter coat can conceal both thin/fat horses, so a simplified BCS score was used.	Inspect and assess fat/muscle from side on neck, shoulder, ribs, back and pelvis Inspect and assess fat deposits on tail bone/caudal vertebra, shape of croup, visibility of spine and hip bone Assess BCS on overall body condition	0—Very poor 1—Poor 2—Moderate 3—Good 4—Fat 5—Very fat *Each body region assessed which, when summed and averaged, provided an overall score* Thin Acceptable Fat
Cresty Neck Score: *Adapted from Carter* et al. *[28]*	Inspect neck from poll to withers	Inspect neck crest fatness	No visible crest Slightly visible crest Noticeable crest Enlarged and thickened crest Grossly enlarged and thickened crest Very large crest; permanently droops to one side
Hair Coat Condition: *Adapted from Animal Welfare Indicators (AWIN) protocol for horses [29]*	Seasonal coat pattern considered during assessment. Severity of negative muddy coat condition was based on a deviation from an accepted health and welfare standard, i.e., horses had limited control over the time they spent in mud in their environment or whether mud was the only option available to them	Inspect whole horse for hair/coat condition	Healthy (Sleek/Glossy) Unhealthy (Dull/Dry) Muddy (positive, e.g., from rolling) Muddy (negative, e.g., from environment)
Generalised Skin Conditions: *Adapted from Welfare Monitoring System (WMS) for horses [30]*	Areas assessed: around eyes, muzzle, back, shoulder, ears, legs	Inspect horse for skin conditions (sunburn, rain scald) visible on the horse’s body only	No evidence of skin conditions Evidence of skin conditions
Skin Irritation on Lower Legs/Pastern: *Adapted from WMS for horses [30]*	Skin irritation visible	Inspect lower legs/pastern for skin irritation indicative of dermatitis, mud fever or mites	No evidence of skin irritation Inflammation, redness, flakes in pastern Swelling/scabs around pastern
Body Lesions: *Adapted from WMS for horses [30]*	Area assessed: muzzle, head, neck, shoulders, mid-section, hind quarters and legs	Inspect and record number of lesions in each area *Only lesions > 1 cm ^2^ area or* *over 4 cm long were included*	None Hairless spot/scar Swollen spot Superficial/healed lesion Injury minor cut through skin Open lesion Deep lesion
Hairless Patches: *Adapted from AWIN [29]*	Areas assessed: muzzle, head, neck, shoulder, midsection, hindquarters and legs	Inspect for hair loss with undamaged skin on each area Record number of hairless patches in each area	No evidence of hairless patches Evidence of hairless patches Number of hairless patches
Swollen Tendon/Joints: *Adapted from AWIN [29]*	Leg location of swollen tendons/joints: near fore (front left), off fore (front right), near hind (back left), off hind (back right)	Inspect for swollen tendons/joints on the following areas: elbow, carpus, fetlock, stifle and hock. Record number in each area	No evidence of swollen tendons/joints Evidence of swollen tendons/joints Number of swollen tendons/joints
Discharge and Coughing: *Adapted from AWIN [29] and Standardised equine-based welfare assessment tool (SEBWAT) [31]*	Nasal discharge Clear watery, discharge Unilateral thick white/ yellow discharge Bilateral thick white/yellow discharge Unilateral dried, crusted Ocular discharge: Clear watery, discharge Thick white/yellow discharge Dried, crusted, discharge Cough	Assess both nostrils and eyes for discharge Cough: evaluate the horse at rest (5 min)	No evidence of discharge and coughing Evidence and type of discharge Evidence of coughing
Heat Stress: *Created for this study*	Heat stress defined as: sweating, flared nostrils, increased respiratory rate, increased respiratory depth, head nodding, apathy	Assess for heat stress: *The presence of four or more of the criteria determined that heat stress was an issue*	Absence of heat stress Evidence of heat stress
Hoof Condition: *Adapted from AWIN and WMS for horses* *[29,30]*	For conditions (other than growth rings), limb location of hoof conditions was recorded as follows: near fore (front left), off fore (front right), near hind (back left), off hind (back right)	Inspect each hoof for the following: shod, hoof neglect *, growth rings, cracks/breakages, abscess, long toe, backed up toe imbalance/twist and club foot	No evidence of hoof conditions Evidence of hoof conditions * Hoof neglect was defined as the hoof having had little or no recent care while the above- mentioned hoof conditions could still be present in the absence of hoof *neglect.*
Resource measures			
Housing Management: *Adapted from AWIN and WMS for horses [29,30]*	Housing management classified as follows: Group (free range) Individual (free range) Stabled Tethered	Assess the type of housing management that horses are based in	Evaluate housing management
Shelter: *Adapted from Mullan* et al. *[32]*	Shelter available: Mud free/dry standing area No shelter available Muddy/wet standing area	Assess environment for shelter from rain, sun, strong winds and record	Record type of shelter available
Water Availability: *Adapted from AWIN* *[29]*	Water availability classified as follows: Available Evidence of availability Unavailable	Assess whether water is available or not at time of assessment *Number of waterpoints and cleanliness of water was also recorded*	Water available Evidence of water availability Water unavailable
Behavioural measures			
Abnormal Behaviours: *Adapted from WMS for horses* *[30]*	Stereotypies: Crib biting, Wind sucking, Weaving, Pacing Walking on tether line	Assess for evidence of stereotypies and record type of stereotypic behaviour observed	No evidence of stereotypies Evidence of stereotypies
Voluntary Animal Approach: *Adapted from AWIN, WMS for horses and SEBWAT* *[29,30,31]*	Friendly response (horse moves towards researcher and sniffs hand) Negative non-reactive response (horse is apathetic, dull and has no interest in approaching researcher) Negative reactive response (horse anxious, frightened, moves away, turns head away, ears flat back, bites or kicks)	Wait for horse to approach *Maximum test time of 3 min from a distance of 1.5 m.*	Record horse’s response to presence of researcher
Qualitative Behaviour Assessment (QBA): *Adapted from Wemelsfelder* *[19]*	The visual analogue scale (VAS) ranged from ‘minimum’, (behavioural expression was absent) to ‘maximum’, (behaviour expression prevalent)	Observe each horse for expression of QBA terms *(as outlined above)*	Individual horses were scored by placing a mark on the scale at a point between minimum and maximum on each of the QBA terms that best reflected the strength of the horse’s expression for each of these terms

**Table 3:** Ordinal logistic regression predicting horse body condition (*n* = 101). Significant results are bolded (*p* < 0.05), degrees of freedom = 1.

Predictor	B	S.E.	Wald	*p*	Exp (B)	95% C.I. for EXP (B)	
						Lower	Upper
Threshold							
BCS = 1.00	−2.500	2.2202	1.268	0.26	0.082	0.001	6.370
BCS = 2.00 (Moderate	−0.096	2.2140	0.002	>0.96	>0.909	0.012	4.657
BCS = 3.00 (Good)	5.533	2.2811	5.884	0.01	252.934	2.893	115.193
Age group < 4	−1.458	0.6749	4.664	**0.03**	0.233	0.062	0.874
Age group > 15	−1.808	2.2348	0.654	0.41	0.164	0.002	13.100
Tethered (not tethered)	0.153	0.5763	0.071	0.79	1.165	0.377	3.606
Poor coat condition = 0.00	0.617	0.8233	0.561	0.45	1.852	0.369	9.301
Hairless patches = 0.00	0.753	0.7656	0.968	0.32	2.124	0.474	9.526
Generalised skin cond = 0.00	−3.014	1.2330	5.975	**0.01**	0.049	0.004	0.550
Skin irritation lower leg pastern = 0.00	3.671	1.3955	6.921	**0.01**	39.302	2.550	60.770
Body lesions = 0.00	−0.486	0.6557	0.550	0.45	0.615	0.170	2.223
Swollen joints = 0.00	0.321	1.3310	0.058	0.81	1.378	0.101	18.713
Not shod = 0.00	−0.504	0.5841	0.745	0.38	0.604	0.192	1.898
Signs of hoof neglect = 0.00	1.964	0.7762	6.399	**0.01**	7.124	1.556	32.617
Voluntary approach (friendly) = 0.00	1.096	0.7296	2.255	0.13	2.991	0.716	12.498

**Table 4:** Binomial logistic regression predicting likelihood of the presence of hoof neglect (0 = absent, 1 = present), (*n* = 101). Significant results are bolded (*p* < 0.05), degrees of freedom = 1.

Predictor	B	S.E.	Wald	*p*	Exp (B)	95% C.I. for EXP (B)	
						Lower	Upper
BCS (median)	−1.484	0.657	5.100	**0.02**	0.227	0.063	0.822
Age	−0.006	0.080	0.005	0.94	0.994	0.849	1.164
Breed (Cobs)	1.220	0.907	1.811	0.17	3.389	0.573	20.041
Breed (Other)	1.040	1.060	0.963	0.32	2.830	0.354	22.614
Tethered	0.188	0.628	0.090	0.76	1.207	0.352	4.135
Poor coat condition	1.900	0.744	6.515	**0.01**	6.684	1.554	28.748
Generalised skin cond.	1.570	1.483	1.120	0.29	4.807	0.263	87.987
Body lesions	1.505	0.706	4.538	**0.03**	4.504	1.128	17.988
Shod	−0.096	0.672	0.021	0.88	0.908	0.243	3.389
Voluntary approach (friendly)	−2.257	0.678	11.070	**0.01**	0.105	0.028	0.396
Skin irritation/pastern	0.473	2.241	0.045	0.83	1.605	0.020	129.742
Discharge	2.098	1.544	1.845	0.17	8.148	0.395	168.137
Shelter mud free/dry	0.181	1.312	0.019	0.89	1.198	0.092	15.676
Constant	3.008	2.587	1.352	0.24	20.240		

**Table 5:** Binomial logistic regression predicting likelihood of the presence of hoof cracks/breakages (0 = absent, 1 = present), (*n* = 104). Significant results are bolded *(p* < 0.05), degrees of freedom = 1.

Predictor	B	S.E.	Wald	*p*	Exp (B)	95% C.I. for EXP (B)	
						Lower	Upper
Tethered	−0.449	0.521	0.743	0.38	0.638	0.230	1.772
BCS (median)	−0.967	0.572	2.856	0.09	0.380	0.124	1.167
Poor coat condition	1.191	0.671	3.151	**0.07**	3.290	0.883	12.250
Hairless patches	1.335	0.663	4.061	**0.04**	3.802	1.037	13.934
Generalised skin cond.	0.593	1.346	0.194	0.65	1.810	0.129	25.322
Skin Irritation/pastern	−3.487	1.911	3.331	**0.06**	0.031	0.001	1.294
Body lesions	1.532	0.626	5.986	**0.01**	4.628	1.356	15.795
Swollen joints	0.005	1.361	0.000	0.99	1.005	0.070	14.484
Discharge	0.225	1.674	0.018	0.89	1.252	0.047	33.316
Cough	−21.841	17,591.713	0.000	0.99	0.000	0.000	
Shod	0.210	0.517	0.165	0.68	1.234	0.448	3.403
Shelter mud free/dry	0.282	1.096	0.066	0.79	1.326	0.155	11.363
Water	−1.001	0.699	2.050	0.15	0.368	0.093	1.447
Constant	2.354	2.242	1.102	0.29	10.524		

**Table 6:** Binomial logistic regression predicting likelihood of horse tethering (0 = absent, 1 = present), (*n* = 104). Significant results are bolded (*p* < 0.05), degrees of freedom = 1.

Predictor	B	S.E.	Wald	*p*	Exp (B)	95% C.I. for EXP (B)	
						Lower	Upper
Age	−0.094	0.062	2.289	0.13	0.910	0.805	1.028
Breed (Cobs)	0.438	0.589	0.553	0.45	1.549	0.489	4.914
Breed (Other)	0.981	0.755	1.687	0.19	2.667	0.607	11.719
BCS (median)	−0.261	0.509	0.263	0.60	0.770	0.284	2.088
Shelter	2.051	0.555	13.640	**0.01**	7.778	2.619	23.103
Constant	0.400	1.553	0.066	0.79	1.491		

**Table 7:** Binomial logistic regression predicting likelihood of friendly response (0 = absent, 1 = present) to the human approach test on predicting variables (*n* = 104). Significant results are bolded *(p* < 0.05), degrees of freedom = 1.

Predictor	B	S.E.	Wald	*p*	Exp (B)	95% C.I. for EXP (B)	
						Lower	Upper
Age	0.134	0.119	1.271	0.26	1.143	0.906	1.443
Breed (Cobs)	−1.634	1.51	1.171	0.27	0.195	0.01	3.765
Breed (Other)	−2.973	1.627	3.34	0.06	0.051	0.002	1.24
Tethered	0.778	0.822	0.897	0.34	2.177	0.435	10.892
BCS (median)	−0.801	0.774	1.073	0.30	0.449	0.098	2.045
Heat stress	0	0	1.046	0.30	1	1	1
Hairless patches	−1.437	0.868	2.737	0.09	0.238	0.043	1.304
Generalised skin cond.	21.962	17,464.15	0	0.99	3.45 × 10^9^	0	.
Skin irritation/pastern	0.617	2.254	0.075	0.78	1.853	0.022	153.52
Body lesions	−0.838	0.84	0.994	0.31	0.433	0.083	2.246
Discharge	19.468	25,382.16	0	0.99	2.85 × 10^8^	0	.
Shod	−0.293	0.746	0.154	0.69	0.746	0.173	3.221
Signs of hoof neglect	−3.108	1.371	5.136	**0.02**	0.045	0.003	0.657
Presence/hoof conditions	0.851	1.254	0.461	0.49	2.342	0.201	27.329
Shelter	−0.335	0.872	0.148	0.70	0.715	0.129	3.949
Shelter muddy/wet	19.714	13,833.5	0	0.99	3.64 × 10^8^	0	.
Water	0.333	0.987	0.114	0.73	1.395	0.201	9.661
Constant	6.003	3.237	3.439	0.06	404.82		

**Table 8:** QBA descriptors in the two main dimensions (PC1, PC2) representing mood and energy and their loadings. The strongest loadings across both dimensions are bolded.

Descriptor	PC1	PC2
Nervous	**−0.792**	0.311
Timid	**−0.761**	0.059
Sociable	**0.678**	0.484
Settled	**0.874**	−0.094
Afraid	**−0.789**	0.298
Good Form	**0.890**	0.065
Laid Back	**0.855**	−0.160
Inquisitive	0.406	**0.728**
Lively	0.139	**0.555**
Down	**−0.376**	−0.061
Aggressive	−0.252	**0.372**
Friendly	**0.940**	0.113
Bored	**−0.427**	0.026
Pushy	−0.057	**0.694**

## Data Availability

The data presented in this study are available on request from the corresponding author.
